# Foreign RNA Induces the Degradation of Mitochondrial Antiviral Signaling Protein (MAVS): The Role of Intracellular Antiviral Factors

**DOI:** 10.1371/journal.pone.0045136

**Published:** 2012-09-17

**Authors:** Fei Xing, Tomoh Matsumiya, Koji Onomoto, Ryo Hayakari, Tadaatsu Imaizumi, Hidemi Yoshida, Mitsutoshi Yoneyama, Takashi Fujita, Kei Satoh

**Affiliations:** 1 Department of Vascular Biology, Institute of Brain Science, Hirosaki University Graduate School of Medicine, Hirosaki, Japan; 2 Division of Molecular Immunology, Medical Mycology Research Center, Chiba University, Chiba, Japan; 3 Laboratory of Molecular Genetics, Institute for Virus Research, Kyoto University, Kyoto, Japan; 4 Laboratory of Molecular Cell Biology, Graduate School of Biostudies, Kyoto University, Kyoto, Japan; University of Washington, United States of America

## Abstract

Mitochondrial antiviral signaling protein (MAVS) is an essential adaptor molecule that is responsible for antiviral signaling triggered by retinoic acid-inducible gene-I (RIG-I)-like receptors (RLRs), leading to the induction of type I interferon in innate immunity. Previous studies have shown that certain viruses evade the innate immune response by cleaving the MAVS protein. However, little is known about how MAVS is regulated in response to foreign RNA, including both single-stranded (ss) and double-stranded (ds) RNA, because most previous reports have shown that the cleavage of MAVS is executed by proteases that are induced or activated by the invading RNA viruses. Here, we report that MAVS mRNA is degraded in response to polyinosinic-polycytidylic acid (polyI:C), a synthetic dsRNA, in A549 cells. RNA interference (RNAi) experiments revealed that both ssRNA- and dsRNA-associated pattern-recognition receptors (PRRs) were not involved in the degradation of MAVS mRNA. Foreign RNA also induced the transient degradation of the MAVS protein. In the resting state, the MAVS protein was protected from degradation by interferon regulatory factor 3 (IRF3); moreover, the dimerization of IRF3 appeared to be correlated with the rescue of protein degradation in response to polyI:C. The overexpression of MAVS enhanced interferon-β (IFN-β) expression in response to polyI:C, suggesting that the degradation of MAVS contributes to the suppression of the hyper-immune reaction in late-phase antiviral signaling. Taken together, these results suggest that the comprehensive regulation of MAVS in response to foreign RNA may be essential to antiviral host defenses.

## Introduction

The antiviral defense system consists of innate and adaptive immunity. The innate immune system is the initial reaction of mammalian cells against invading pathogens. The recognition of pathogen-associated molecular patterns (PAMPs) on the surface of the pathogens by pattern recognition receptors (PRRs) is the key to the activation of the inherent innate immune response [1]. Among the PRRs, retinoic acid-inducible gene-I (RIG-I)-like receptors (RLRs), including RIG-I [2], melanoma differentiation-associated gene-5 (MDA-5) [3] and laboratory of genetics and physiology 2 (LGP2) [4], are expressed in various types of cells. The RLRs are expressed in the cytoplasm [5]. After the recognition of viral RNA, both RIG-I and MDA-5 expose their N-terminal caspase recruitment and activation domains (CARDs). The exposed CARDs interact with a downstream adaptor molecule, mitochondrial antiviral signaling protein (MAVS) [6], which is also known as virus-induced signaling adaptor (VISA) [7], interferon (IFN)-β promoter stimulator-1 (IPS-1) [8] and caspase activation and recruitment domain adaptor inducing IFN-β (Cardif) [9]. MAVS then activates TANK-binding kinase 1 (TBK1) and IκB kinases (IKKs), resulting in the activation of interferon regulatory factor 3 (IRF3) and nuclear factor-κB (NF-κB) [10]. The coordinated activation of IRF3 and NF-κB eventually induces the secretion of type I IFNs and pro-inflammatory cytokines that have antiviral activities [11].

The MAVS protein is composed of an N-terminal CARD, which is required for RLRs signaling; an internal proline-rich region; and a C-terminal transmembrane (TM) domain. Structural analysis has revealed that the CARD of MAVS shares high sequence similarity with the CARDs of RIG-I and MDA-5, suggesting that MAVS binds to homotypically RIG-I or MDA-5 through this domain [12]. Notably, the members of the tumor necrosis factor receptor associated factor (TRAF) family are necessary for MAVS-dependent antiviral signaling [10].

In the antiviral state, a direct interaction between the TRAF domain of TRAF and the proline-rich region within MAVS occurs [7], [13]. The TM domain targets itself to the mitochondrial outer membrane [6]. The distribution of MAVS in the mitochondria is vital for its role in signaling, as dissociation from the mitochondria resulting from the deletion of the TM domain results in the ablation of signaling [14]. Certain viruses escape from PRR-dependent antiviral responses by cleaving MAVS from the mitochondria membrane [15]. To date, MAVS has been shown to be degraded by the three following processes: the direct cleavage of MAVS by viral proteases or their precursors [14]–[18]; the cleavage of MAVS by certain members of the caspase family, which is observed during apoptosis [19]–[21]; and the ubiquitination-mediated proteasomal degradation of MAVS [22]–[24]. However, little is known about the regulation of MAVS mRNA.

In this study, we found that both ssRNA and dsRNA induced the degradation of MAVS mRNA in A549 human lung cancer cells. We also found that the silencing of IRF3, a crucial downstream molecule in the MAVS-dependent antiviral signaling pathway [2], facilitates the degradation of the MAVS protein, whereas the silencing of IRF3 does not affect the level of MAVS mRNA. Our findings address the comprehensive regulation of MAVS in relation to antiviral signaling.

## Materials and Methods

### Cell Culture and Treatment

A549 and 293 cells (JCRB, Japan) were maintained in a 5% CO_2_ atmosphere at 37°C in Dulbecco’s modified Eagle’s medium (DMEM) (Sigma-Aldrich, St. Louis, MO) supplemented with 10% fetal bovine serum (FBS) (Perbio Science, Switzerland) and antibiotics. When indicated, cycloheximide (10 µg/ml) was added to the culture medium 1 h before polyinosinic-polycytidylic acid (polyI:C) (Sigma-Aldrich) transfection.

### Analysis of 5-ethynyl Uridine (EU)-labeled Transcripts

Endogenously transcribed mRNA was labeled using a Click-iT Nascent RNA Capture kit (Invitrogen) under the conditions recommended by the manufacturer. Briefly, A549 cells were pulsed with 0.2 mM EU for 24 h prior to polyI:C transfection. At the indicated time, total RNA was isolated and subjected to a copper-catalyzed click reaction with azide-modified biotin. The nascent transcripts were then captured on streptavidin magnetic beads and were used for cDNA synthesis using the Superscript VILO cDNA synthesis kit (Invitrogen), followed by analysis with quantitative RT-PCR.

### Plasmid and siRNA

cDNA encoding full-length MAVS was amplified from cDNA isolated from HeLa cells using Phusion DNA polymerase (Finnzymes, Keilaranta, Finland) and the primers SalI-MAVS-F (5′-ATTCGgtcgacCATGCCGTTTGCTGAAGAC-3′) and NotI-MAVS-R (5′-CGAgcggccgcCTAGTGCAGACGCCGCCG-3′). The amplified product was inserted into the SalI and NotI sites of a mammalian expression vector, pCMV-myc (Clontech, Mountain View, CA). The DNA construct was analyzed by DNA sequencing. The plasmid DNA was purified using a plasmid purification column (Qiagen, Hilden, Germany).

SiRNAs against TLR3 (SI102655156), RIG-I (SI102657403), MDA-5 (SI03649037) and IRF3 (SI03117359) and non-silencing control siRNA were purchased from Qiagen.

### Generation of Single-stranded (ss) and Double-stranded (ds) RNA

To generate synthetic ss- and dsRNA, the β-lactamase (*bla*) gene was chosen as an RNA template, and the *bla* gene from pcDNA3.1/Zeo (+) (Invitrogen) was cloned with the primers T7-bla-F (5′-GGATCCTAATACGACTCACTATAGGttaccaatgcttaatcag-3′) and bla-R 5′-ggatgagtattcaacatttc-3′) to yield sense ssRNA or with the primers bla-F (5′-ggttaccaatgcttaatcag-3′) and bla-R 5′-GGATCCTAATACGACTCACTATAGGatgagtattcaacatttc-3′) to yield antisense ssRNA. Following DNA purification, both ssRNAs were synthesized using in vitro transcription with a T7 RiboMAX Large Scale RNA Production kit (Promega, Madison, MI). The ssRNAs were then annealed to generate dsRNA. The annealed dsRNA was assessed by agarose gel electrophoresis.

### Transfection

Transient transfections of A549 cells were performed as previously reported [25]. Briefly, the cells were seeded at a density of 1.8×10^5^ cells per well in 12-well culture plates or at a density of 3.7×10^5^ cells in 35 mm dishes for 16–20 h before transfection to reach a 70–80% confluence.

To overexpress exogenous MAVS, the cells were transfected with a pCMV-myc-MAVS or pCMV-myc expression vector using Lipofectamine 2000 (Invitrogen) and incubated for 24 h. To introduce foreign RNA (polyI:C, ssRNA or dsRNA), 293 cells or A549 cells were transfected using Lipofectamine 2000 for the indicated period of time. RNA interference (RNAi) was induced via transfection with gene-specific siRNAs or control siRNA using Lipofectamine RNAiMAX (Invitrogen), following the manufacturer’s instructions.

### Virus Infections

Sendai virus (SeV) and new castle disease virus (NDV) stocks were grown in the allontonic cavities of 11-day-old embryonated eggs. Influenza A virus (IAV) ΔNS1, originally produced by Dr. A. Garcia-Sastre (Mount Sinai School of Medicine, USA) and provided by Dr. S. Akira (Osaka University, Japan), were grown in the allantoic cavities of 9-day-old embryonated eggs. Cells were treated with the culture medium (‘mock-treated’) or infected with the viruses in serum-free and antibiotic-free medium. After an adsorption period for 1 h at 37°C, the inoculum was removed and replaced with fresh medium.

### Quantitative RT-PCR

Total RNA was extracted from the cells using an Illustra RNAspin Mini RNA Isolation Kit (GE Healthcare, Piscataway, NJ). Total RNA (500 ng) served as a template for single-strand cDNA synthesis in a reaction using an oligo(dT) primer and M-Mulv reverse transcriptase (Invitrogen) under the conditions indicated by the manufacturer. A CFX96 real-time PCR detection system (Bio-Rad, Hercules, CA) was used for the quantitative analyses of IFN-β, RIG-I, MDA5, MAVS, IRF3, Toll-like receptor 3 (TLR3), 18S rRNA, and glyceraldehyde-3-phosphate dehydrogenase (GAPDH). The sequences of the primers were as follows:

IFN-β-F (5′-CCTGTGGCAATTGAATGGGAGGC-3′),

IFN-β-R (5′-CCAGGCACAGTGACTGTACTCCTT-3′),

RIG-I-F (5′-GTGCAAAGCCTTGGCATGT-3′),

RIG-I-R (5′-TGGCTTGGGATGTGGTCTACTC-3′),

MDA5-F (5′-GTTGAAAAGGCTGGCTGAAAAC-3′),

MDA5-R (5′-TCGATAACTCCTGAACCACTG-3′),

18S rRNA-F (5′-ACTCAACACGGGAAACCTCA-3′),

18S rRNA-R (5′-AACCAGACAAATCGCTCCAC-3′),

GAPDH-F (5′-GCACCGTCAAGGCTGAGAAC-3′),

GAPDH-R (5′-ATGGTGGTGAAGACGCCAGT-3′),

MAVS-F (5′-ATAAGTCCDGAGGGCACCTTT-3′),

MAVS-R (5′-GTGACTACCAGCACCCCTGT-3′),

IRF3-F (5′-TACGTGAGGCATGTGCTGA-3′),

IRF3-R (5′-AGTGGGTGGCTGTTGGAAAT-3′),

TLR3-F (5′-CTCAGAAGATTACCAGCCGCC-3′) and

TLR3-R (5′-CCATTATGAGACAGATCTAATG-3′).

The amplification reactions were performed with SsoFast EvaGreen Supermix (Bio-Rad) according to the manufacturer’s specifications. The amplification conditions were as follows: heat for 30 sec at 98°C, followed by heating consecutively at 98°C and 58°C for 5 sec each for 40 cycles. After amplification was complete, a melting curve was generated by slowly heating from 65°C to 95°C at 0.5°C increments with 5 sec per step, with the continuous monitoring of the fluorescence. The melting curves and quantitative analysis of the data were performed using a CFX manager (BioRad).

### Immunoblot Analyses

After two washes with phosphate-buffered saline (PBS), the cells were lysed in hypotonic lysis buffer [10 mM Tris (pH 7.4), 100 mM NaCl, 1.5 mM MgCl_2_, 0.5% NP-40] containing 0.2% protease inhibitors. The lysate was cleared by centrifugation at 6,000 rpm for 15 min at 4°C. Ten micrograms of the cell lysate was subjected to electrophoresis on a 10% SDS-polyacrylamide gel. The proteins were then transferred to polyvinylidene fluoride (PVDF) membranes (Millipore, Billerica, MA), which were then blocked for 1 h at room temperature in TBST buffer [20 mM Tris (pH 7.4), 150 mM NaCl, 0.1% Tween 20] containing 5% nonfat dry milk (blocking buffer). The membranes were incubated overnight at 4°C with one of the following primary antibodies: mouse anti-RIG-I, mouse anti-Cardif (MAVS) (Enzo Life Sciences, Miami, FL), rabbit anti-IRF3 (IBL, Japan), mouse anti-GAPDH (BioLegend, San Diego, CA) or rabbit anti-β-actin (Sigma-Aldrich). After five washes with TBST, the membranes were further incubated for 1 h at room temperature with bovine anti-rabbit (Santa Cruz Biotechnology, Santa Cruz, CA) or Zymax anti-mouse IgG antibody (Invitrogen) coupled with HRP at a 1∶10,000 dilution in blocking buffer. The washes were repeated using TBST, and then the immunoreactive bands were visualized using the Luminata Crescendo Western HRP Substrate (Millipore).

For native PAGE analysis, A549 cells were harvested in native lysis buffer [50 mM Tris-Cl (pH 8.0), 1% NP40, 150 mM NaCl]. After centrifugation at 12,000 rpm for 10 min at 4°C, the lysates were subjected to native PAGE electrophoresis, as previously reported [26].

### Immunofluorescence Analyses

Immunofluorescence stains were performed as reported previously [27]. Briefly, A549 cells were grown on glass coverslips and then fixed with 4% formaldehyde for 20 min, permeabilized with 0.1% Triton X-100 for 10 min and blocked with 3% BSA for 1 h. The cells were then incubated for 1 h with mouse monoclonal anti-MAVS and rabbit anti-IRF3 antibodies. After a washing step, the cells were incubated with Alexa 488-conjugated anti-mouse IgG and anti-Alexa 555-conjugated anti-rabbit IgG (Invitrogen). The cells were mounted in Prolong gold antifade reagent (Invitrogen), and the localizations of MAVS and IRF3 were visualized by confocal laser scanning microscopy (C1si, Nikon, Japan).

**Figure 1:**
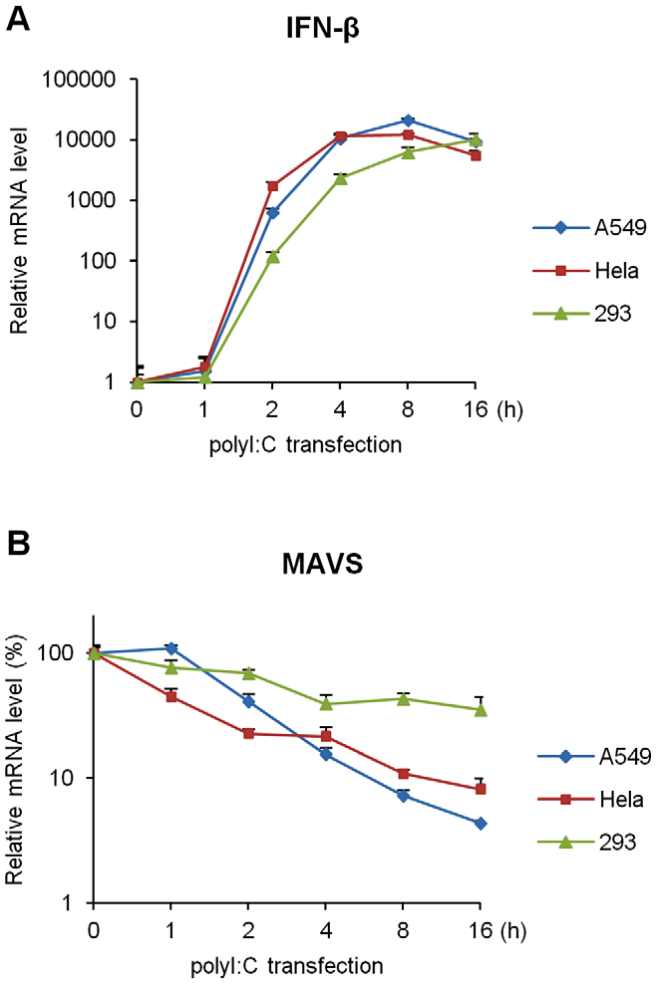
Time course of the down-regulation of MAVS by polyI:C. A549 (blue), HeLa (red) and 293 (green) cellswere transfected with polyI:C (500 ng/well). Quantitative RT-PCR for IFN-β and MAVS was performed. Data are presented as the mean ± SD of three independent experiments.

### Time-lapse Microscopy

A549 cells transfected with polyI:C (250 ng/well) or control untransfected cells (control) were placed in a 5% CO_2_ atmosphere at 37°C microscope chamber and observed with a x20 long-working distance objective lens mounted on an Nikon Ti-E inverted microscope. Images were acquired every 15 min for 16 h using a CoolSNAP HQ2 camera (Roper).

## Results

### PolyI:C Decreases the Expression Level of MAVS mRNA

Several studies have reported that the MAVS protein is degraded by both viral and cellular proteins [15], [18], [22], [23]. However, few studies have investigated the regulation of MAVS mRNA. Moreover, no study has shown the effect of foreign RNA, including viral RNA, on the level of MAVS mRNA. Therefore, our first objective was to determine whether the level of MAVS mRNA is altered in response to the foreign RNA. We initially found that the introduction of polyI:C, a synthetic viral double-stranded RNA analog, significantly down-regulated the expression of MAVS mRNA in A549 cells. The level of MAVS mRNA decreased in a time-dependent manner (Fig. 1A). We next asked whether this phenomenon is limited in A549 cells. In HeLa cells, MAVS mRNA exhibited similar expression pattern during the exposure of polyI:C. In 293 cells, however, the level of MAVS mRNA was relatively stable (<4 h) even by the exposure of polyI:C and it gradually decreased with slow kinetics (Fig. 1A). We confirmed the mRNA expression of IFN-β was upregulated by the introduction of polyI:C in any type of cells we used in this study (Fig. 1B); moreover, cytotoxic activity was observed following the transfection of polyI:C in A549 cells (Movie S1). These results indicated that antiviral signaling is activated during the down-regulation of MAVS mRNA.

**Figure 2:**
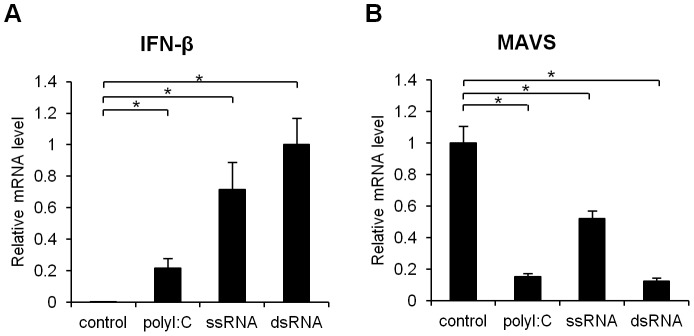
Both ds- and ss-RNA induce MAVS mRNA degradaion. Four hours after the transfection, the mRNA levels of IFN-β (A) and MAVS (B) were determined using quantitative RT-PCR. Data are presented as the mean ± SD of three independent experiments. *, p<0.01.

To determine if the down-regulation is specific to polyI:C, we next synthesized ssRNA and dsRNA from the *bla* gene and transfected these RNAs into A549 cells. In addition to the introduction of polyI:C, the introduction of either ssRNA or dsRNA induced the expression of IFN-β (Fig. 2A), indicating that our system reflected an antiviral state. The down-regulation of MAVS mRNA was also observed when the cells were transfected with either ssRNA or dsRNA (Fig. 2B).

### Effects of RNA Viruses on the Level of MAVS mRNA

**Figure 3:**
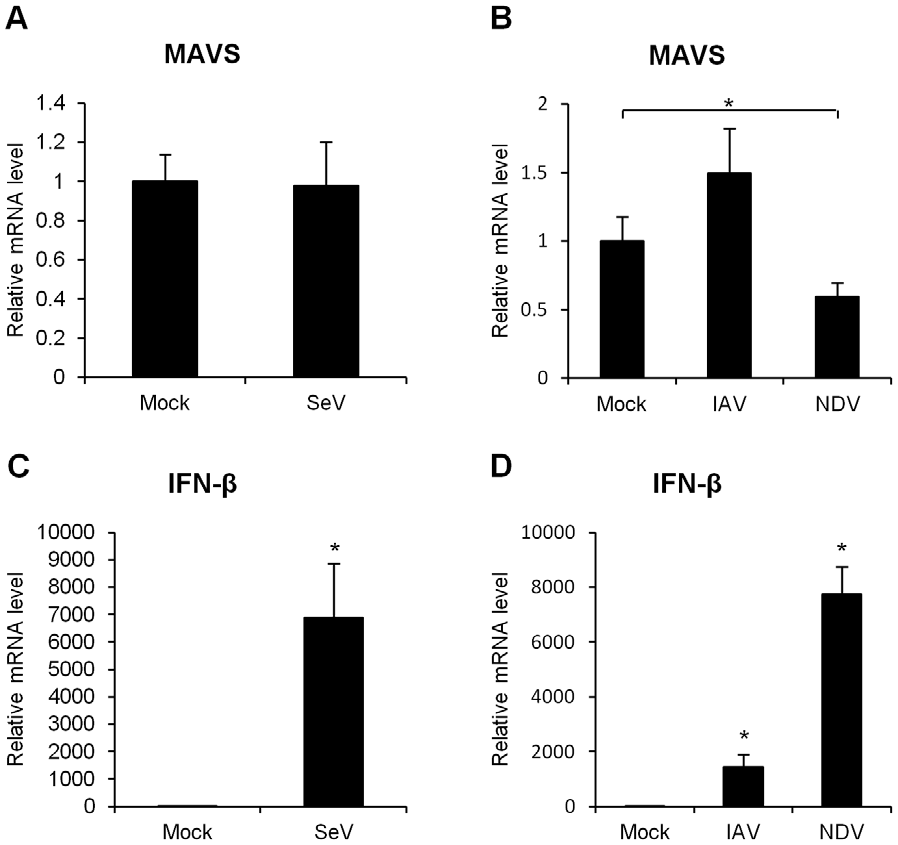
Influence of MAVS mRNA on viral infection. (A, C) HeLa cells were infected with mock-treated or SeV for 9 h. (B) HeLa cells were infected with mock-treated, IAV, or NDV. Quantitative RT-PCR for (A, B) MAVS and (C,D) IFN-β was performed. Data are presented as the mean ± SD of three independent experiments. *, p<0.01.

Either SeV or IAV infection did not alter the level of MAVS mRNA in HeLa cells (Fig. 3A, B). In contrast, NDV infection significantly down-regulated the level of MAVS mRNA (Fig. 3B) These results suggested that the effect of viral infection on MAVS mRNA down-regulation differs among RNA viruses.

### PolyI:C Destabilizes MAVS mRNA

**Figure 4:**
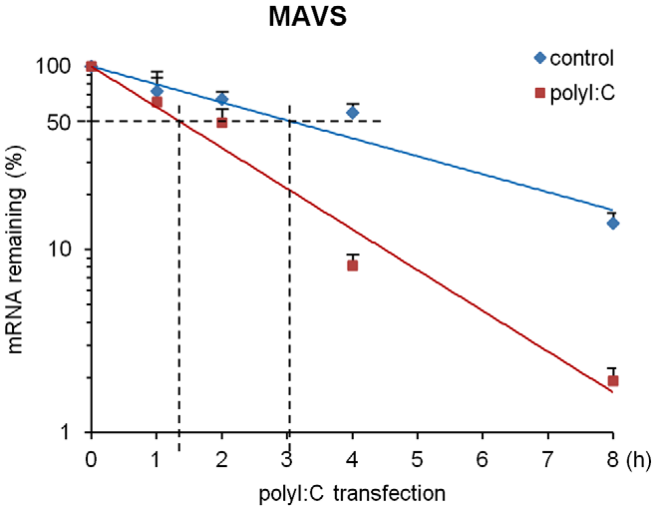
MAVS mRNA decays rapidly in response to polyI:C transfection. A549 cells were pretreated with EU 24 h before polyI:C transfection. At the indicated time points after transfection, EU-labeled mRNA was isolated and quantified using quantitative RT-PCR. Data are presented as the mean of three independent experiments.

We next determined whether the polyI:C-mediated down-regulation of MAVS mRNA was due to a change in mRNA stability. Actinomycin D, an RNA synthesis inhibitor, is widely used for the study of mRNA stability. However, the treatment of A549 cells with actinomycin D in the presence of polyI:C rapidly induced cell death because of the cytotoxic activity of actinomycin D. To exclude such an unexpected effect of both actinomycin D and polyI:C on the transcription of MAVS mRNA, we utilized a recently developed tool for studying mRNA stability. 5-Ethynyl uridine (EU) added to the culture medium was incorporated into cells, resulting in its incorporation into *de novo* synthesized mRNA. By replacing the culture medium with fresh EU-free medium, which ends the incorporation of EU into *de novo* synthesized mRNA, we were able to monitor mRNA decay. We added EU to the culture medium and pre-incubated A549 cells for 24 h. We then replaced the EU-containing medium with fresh EU-free medium immediately prior to the polyI:C transfection and monitored the level of MAVS mRNA. As shown in Fig. 4, MAVS mRNA in A549 cells underwent relatively slow decay. The introduction of polyI:C resulted in the rapid decay of MAVS mRNA. The half-lives of MAVS mRNA in control cells and cells transfected with polyI:C were calculated to be 81 min and 183 min, respectively.

### RLRs and TLR3 are not Involved in the Regulation of MAVS mRNA

**Figure 5:**
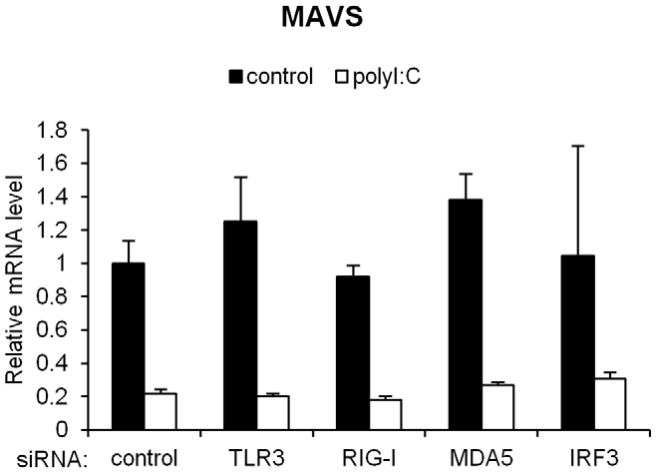
Involvement of TLR3, RLRs and IRF3 in the polyI:C-induced degradation of MAVS mRNA. A549 cells were transfected with siRNA against TLR3, RIG-I, MDA-5 or IRF3 or with a control (scrambled) siRNA. Forty-eight hours after transfection, polyI:C was introduced into the cells for 4 h. The MAVS mRNA level was analyzed using quantitative RT-PCR. Data are presented as the mean ± SD of three independent experiments.

Our next goal was to identify the molecule(s) involved in the down-regulation of MAVS mRNA induced by foreign RNA. Because RIG-I, MDA-5 and TLR3 are RNA-sensing PRRs [1], we examined the involvement of those PRRs in the decrease in the MAVS mRNA level in response to the introduction of polyI:C. The silencing of these PRRs by RNAi did not affect the level of MAVS mRNA in cells in either the resting state or the antiviral state (Fig. 5, S1).

### Involvement of IRF3 in the polyI:C-mediated Inhibition of MAVS Protein

**Figure 6:**
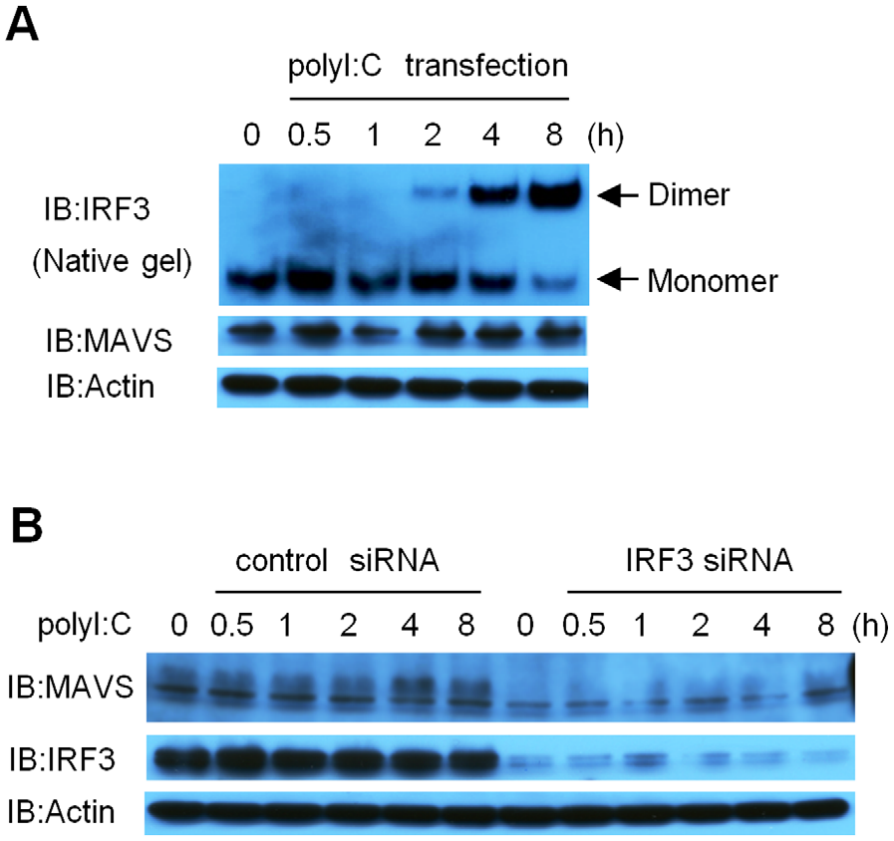
Protective effect of IRF3 on the MAVS protein. (A) A549 cells were transfected with polyI:C and incubated for up to 8 h. (B) Following IRF3 knockdown, A549 cells were transfected with polyI:C. Cell extracts were subjected to either SDS- (A and B) or native PAGE (A) and then blotted with anti-IRF3, anti-MAVS or anti-actin antibodies, as indicated. The results are representative of three independent experiments.

IRF3 is involved in the polyI:C-induced degradation of rRNA [28]. Therefore, we investigated the role of IRF3 in the degradation of MAVS mRNA in response to polyI:C and observed that IRF3 was not involved in the increased degradation of MAVS mRNA in response to polyI:C (Fig. 5, S1). The level of the MAVS protein transiently decreased at 1 h after transfection with polyI:C and gradually recovered to the basal level (Fig. 6A). IRF3 dimerization was observed from 2 h after the transfection. The silencing of IRF3 facilitated the marked inhibition of the MAVS protein in the resting state, whereas the non-silencing control siRNA did not have such an effect (Fig. 6B). Thus, IRF3 knockdown did not influence the kinetics of the MAVS protein in response to polyI:C transfection (Fig. 6B).

### Stability of the MAVS Protein in the Presence of polyI:C

**Figure 7:**
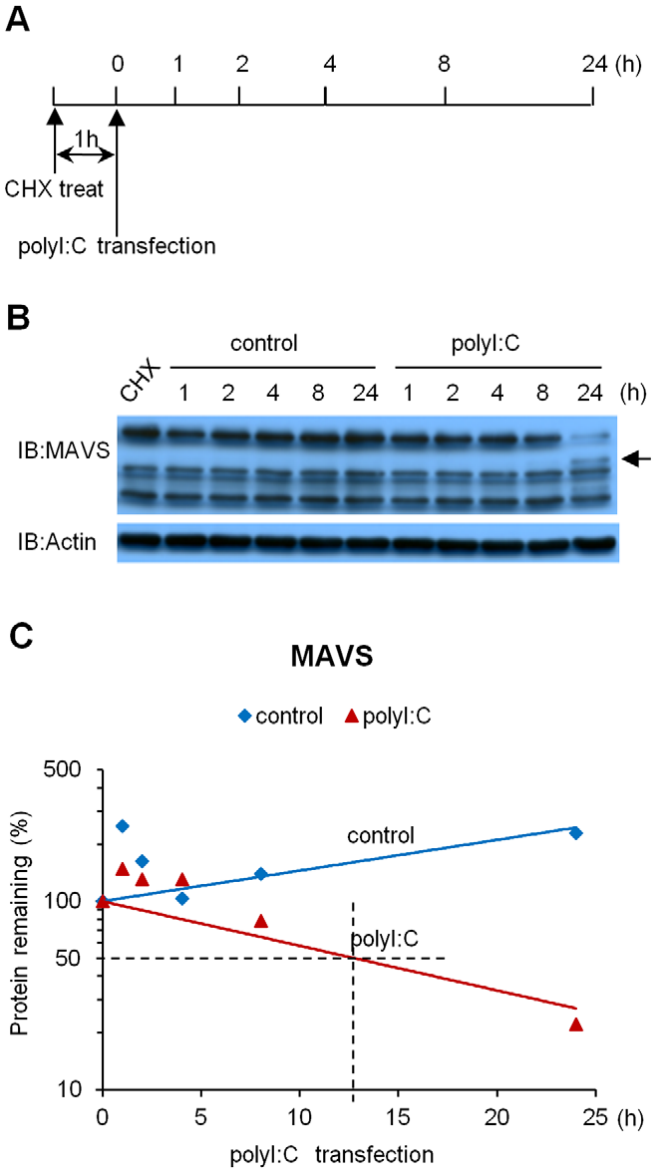
Protein stability of MAVS in response to polyI:C transfection. A549 cells were transfected with polyI:C for 1 h, followed by treatment with cycloheximide (CHX) for up to 24 h (illustrated as (A)). Cell extracts were analyzed by immunoblotting. The intensities of the immunoblot bands were quantified by Image J. Data are presented as the means of three independent experiments (B) or are representative of three independent experiments (C). The arrow indicates the cleaved MAVS protein.

Next, we examined MAVS protein stability in response to polyI:C transfection in A549 cells. Cycloheximide, a protein synthesis inhibitor, was added to the culture medium to stop the *de novo* synthesis of the MAVS protein after transfection with polyI:C. We then followed the decay of the MAVS protein as we did for the decay of MAVS mRNA. In the resting state, the MAVS protein was quite stable (Fig. 7B). Transfection with polyI:C induced the decay of the MAVS protein in A549 cells (Fig.7C).

The estimated half-life for the MAVS protein was approximately 12.7 h after the introduction of polyI:C, whereas the level of protein in the control cells remained stable even at 24 h after the introduction of polyI:C.

### Importance of the Amount of the MAVS Protein to IFN-β Induction in Response to polyI:C

**Figure 8:**
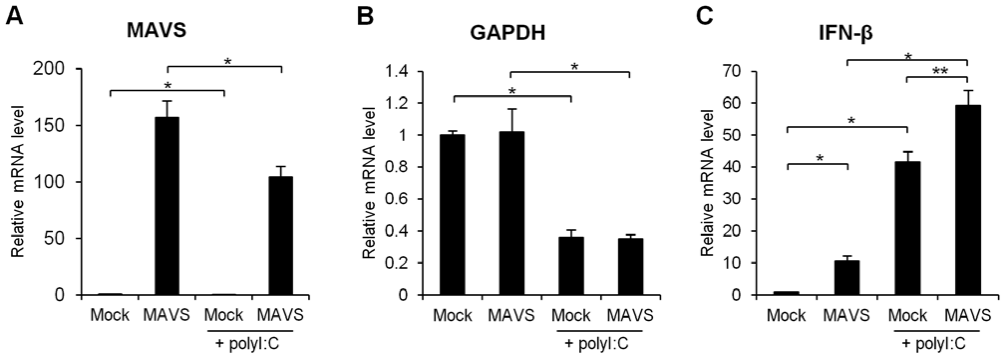
Effect of MAVS overexpression on polyI:C-induced IFN-β expression. A MAVS-expressing plasmid or an empty plasmid (mock) was transfected into A549 cells for 24 h before polyI:C transfection. The mRNA levels of MAVS (A), GAPDH (B) and IFN-β (C) were determined using quantitative RT-PCR. Data are presented as the mean ± SD of three independent experiments. *, p<0.01.

Because MAVS is a crucial molecule in RNA-mediated antiviral signaling, we determined the effect of MAVS degradation on polyI:C-mediated antiviral signaling. To address the potential role of MAVS, exogenous MAVS was overexpressed in A549 cells. The level of MAVS mRNA was significantly up-regulated after transfection with an expression plasmid encoding the full-length MAVS cDNA. Following the introduction of polyI:C, MAVS mRNA in the overexpressing cells was degraded, as endogenous MAVS mRNA was (Fig. 8A), suggesting that poly I:C induced cleavage of the MAVS mRNA within its coding sequence. We noted that MAVS overexpression had no effect on the GAPDH repression induced by polyI:C (Fig. 8B). We found that overexpressed MAVS triggered more dramatic IFN-β induction in response to polyI:C compared with the control (Fig. 8C).

## Discussion

In the present study, we found that MAVS mRNA was degraded in response to the introduction of foreign RNA. Although over 100 articles have been published about MAVS, the transcriptional regulation of MAVS mRNA has not been investigated. Neither type I IFNs nor inflammatory cytokines, including TNF-α and IL-1, alter the expression of MAVS mRNA, whereas these proteins do affect a variety of antiviral molecules, including RIG-I and MDA-5 (Matsumiya T, unpublished data). In the present study, we found that the MAVS mRNA and protein are stable in the resting state. These observations allowed us to speculate that MAVS may be categorized as a so-called housekeeping gene, as another group proposed [22]. A recent study has indicated that housekeeping genes, including 18S rRNA and GAPDH, are highly regulated in chicken embryo fibroblasts during new castle disease virus (NDV) infection [29]. NDV not only down-regulates the expression of GAPDH but also up-regulates the expression of succinate dehydrogenase complex, subunit A (SDHA), another housekeeping gene. These results indicate that the regulation of the expression of housekeeping genes in response to viral infection differs among housekeeping genes. Piechaczyk et al. reported that the levels of GAPDH mRNA are different in various tissues, whereas the transcription rates are similar in those tissues, indicating that GAPDH mRNA is regulated at the post-transcriptional level [30]. In this study, we found that GAPDH mRNA is rapidly degraded following the introduction of dsRNA into the cell, as is MAVS mRNA. Taken together, our results and those of earlier studies indicate that the level of MAVS mRNA is modulated by post-transcriptional regulation.

Viral RNA, a synthetic dsRNA (polyI:C) and 5′-triphosphate ssRNA are recognized by intracellular RNA sensors, including TLR3, RIG-I and MDA-5 [10], [31]. RIG-I and MDA-5 utilize MAVS as an adaptor molecule, whereas TLR3 utilizes Toll-IL-1 receptor domain-containing adaptor inducing IFN-β (TRIF, also known as TICAM-1) [32].

Based on our initial observation that polyI:C mediates the degradation of MAVS mRNA, we asked whether this effect is due to a negative feedback loop involving RIG-I or MDA-5 or to signal crosstalk between MAVS and TLR3. Both ssRNA and dsRNA were able to induce the degradation of MAVS mRNA (Fig. 2B). Because TLR3 senses only dsRNA [33], we excluded the involvement of TLR3 in the degradation of MAVS mRNA in this study. We further observed that RIG-I and MDA-5 do not participate in foreign RNA-mediated MAVS mRNA degradation. This result suggests that the foreign RNA-mediated mRNA degradation of MAVS does not involve PRRs, and the mechanisms remain unclear. dsRNA-dependent protein kinase (PKR) can also recognize dsRNA and exert an antiviral activity via the rapid inhibition of protein synthesis, suggesting an essential role of PKR in responses to viral infection [34]. A recent report has shown that, in addition to dsRNA, 5′-triphosphate-ssRNA can activate PKR in the presence of IFN [35]. In this study, however, no increase in IFN-β expression in response to polyI:C was observed when MAVS mRNA underwent polyI:C-induced mRNA degradation. These results, combined with previous reports, indicate that the degradation of MAVS mRNA is likely independent of PKR.

IRF3, a molecule downstream of MAVS, is one of the most essential molecules in antiviral signaling, particularly in the induction of type I IFN [36]. Uno et al. reported that IRF3 plays a crucial role in rRNA degradation in response to polyI:C-cationic liposome complexes [28]. However, Our results are not sufficient to explain the degradation of MAVS mRNA via IRF3 because the silencing of IRF3 did not alter the half-life of MAVS mRNA following the introduction of polyI:C. Uno et al. also reported that the degradation of rRNA induced by the polyI:C and cationic lipid complex is independent of dsRNA-dependent protein kinase (PKR), RNase L and type I IFN receptor, all of which are involved in the type I IFN-mediated antiviral responses. These researchers finally concluded that an unknown RNase might participate in the degradation of rRNA. In contrast, Sobol et al. reported that transfection with polyI:C induces RNase L-dependent rRNA cleavage in A549 cells [37]. In addition, RNase L has been reported to target mRNAs to ribosomal proteins [38]. When considered in combination with these reports, our results support the hypothesis that foreign RNA activates RNase L, or an unknown RNase, to cleave MAVS within its coding sequence. However, the mechanism of this RNase should be clarified in future studies.

In contrast to the level of MAVS mRNA, we found that the level of MAVS protein is IRF3 dependent. First, it appears that the level of MAVS in resting cells is regulated by constitutively expressed IRF3. Moreover, in the presence of IRF3, MAVS protein is quite stable, as compared with MAVS mRNA (Fig. 6, 7). We observed neither a physical interaction between IRF3 and MAVS (Fig. S2) nor a dimerization of IRF3 (Fig. 6A) in resting cells. The cells lacking IRF3 decreases the level of MAVS protein, even in the resting state, suggesting that the molecule “IRF3” somehow protects MAVS from its protein degradation. Second, the kinetics of the MAVS protein in response to polyI:C transfection appeared to be correlated with that of the dimerization of IRF3; thus, upon transfection of cells with polyI:C, the transient down-regulation of the MAVS protein might be rescued by the activation of IRF3. Interestingly, IRF3 does not alter the half-life of MAVS mRNA in response to polyI:C (Fig. S3). Although whether IRF3 is involved in protein synthesis remains to be elucidated, our results collectively suggest that IRF3 participates in the posttranscriptional regulation of MAVS.

The overexpression of MAVS partially increased polyI:C-induced IFN-β expression. Our study on the protein stability of MAVS showed that polyI:C started to induce the degradation of the MAVS protein from 8 h after the transfection. As has been reported previously, the introduction of polyI:C induces a cytotoxic effect [28], [39]; therefore, we could not demonstrate the effect of MAVS degradation on antiviral signaling, including type I IFN induction, in A549 cells. However, we observed that the overexpression of MAVS increased the level of polyI:C-induced IFN-β mRNA. This result suggested that the inhibition of MAVS might function in antiviral signaling. It is well understood that viruses induce MAVS degradation to evade antiviral innate immunity [40]. In addition, the level of MAVS has been proposed to be tightly regulated to protect cells from the antiviral immune response and excess inflammation [22]. Combining our data with this hypothesis, we speculate that foreign RNA induces MAVS degradation via a negative feedback loop to protect against hyper-immune reactions, at least in the late phase of antiviral signaling.

In summary, our study demonstrated a new mechanism in which MAVS-mediated antiviral immunity is regulated at both the mRNA and protein levels. Although it appears that IRF3 is essential for the protection of MAVS from protein degradation, unknown mechanisms must be required for the degradation of MAVS mRNA.

## Supporting Information

Figure S1 **Effects of RNAi against TLR3, RLRs and IRF3.** A549 cells were transfected with siRNA against RIG-I, MDA5, TLR3, IRF3 or control (scrambled) siRNA. Forty-eight hours after the transfection, the cells were further transfected with polyI:C. Four hours after the additional transfection, total RNA from the cells was extracted and further analyzed by quantitative RT-PCR (upper panels) or by immunoblotting (lower panels)Data are presented as mean ± SD of three independent experiments. *, p<0.01 (upper panels).
(TIF)

Figure S2 **IRF3 does not co-localize with MAVS.** A549 cells were fixed with 4% paraformaldehyde and incubated with anti-MAVS and anti-IRF3antibodies. MAVS and IRF3 proteins were detected with secondary antibody coupled to Alexa 488 (MAVS, green) or Alexa555 (IRF3, red).
(TIF)

Figure S3 **IRF3 does not influence MAVS mRNA decay.** Following the knockdown of IRF3 in A549 cells, polyI:C was transfected and the cells were incubated for up to 8 h. Total RNA from the cells was extracted and further analyzed by quantitative RT-PCR. Data are presented as mean ± SD of three independent experiments.
(TIF)

Movie S1 **Cytotoxicity after transfection of polyI:C.** Time-lapse series showing cytotoxicity following transfection of polyI:C in A549 cells.
(MOV)
