# Supplementary figures and images for: Foreign RNA Induces the Degradation of Mitochondrial Antiviral Signaling Protein (MAVS): The Role of Intracellular Antiviral Factors
Source: PLoS One. 2012 Sep 17;7(9):e45136. doi: 10.1371/journal.pone.0045136 (PMC3444469; doi:10.1371/journal.pone.0045136)

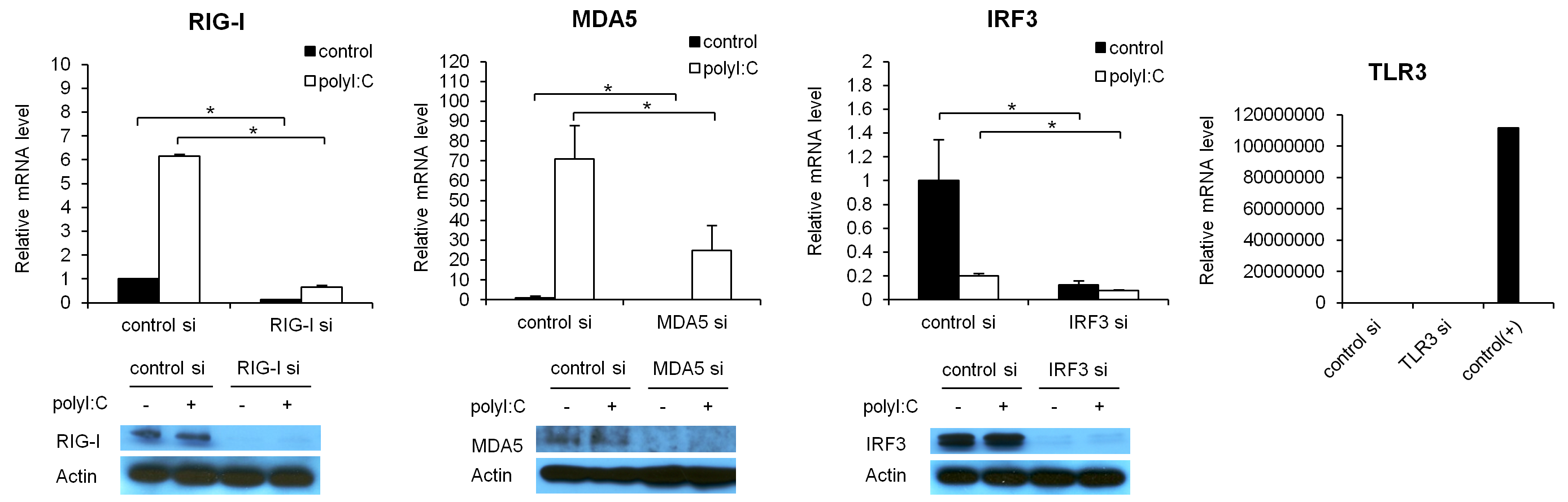

Supplement: Figure S1 — Effects of RNAi against TLR3, RLRs and IRF3. A549 cells were transfected with siRNA against RIG-I, MDA5, TLR3, IRF3 or control (scrambled) siRNA. Forty-eight hours after the transfection, the cells were further transfected with polyI:C. Four hours after the additional transfection, total RNA from the cells was extracted and further analyzed by quantitative RT-PCR (upper panels) or by immunoblotting (lower panels)Data are presented as mean ± SD of three independent experiments. *, p<0.01 (upper panels). (TIF) [file pone.0045136.s001.tif]

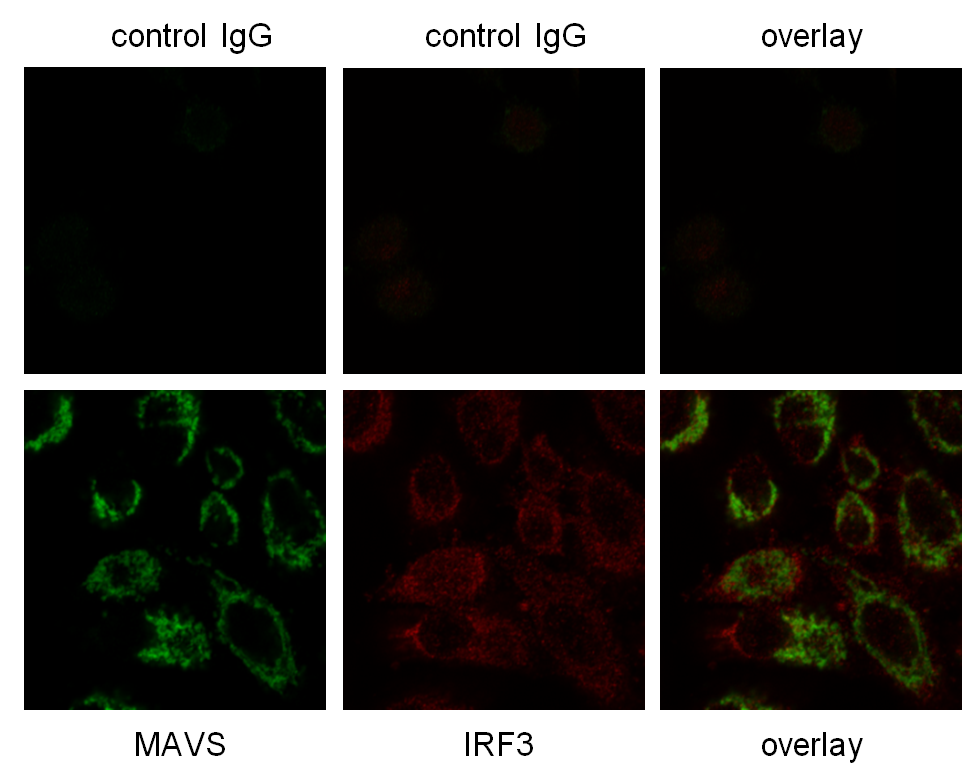

Supplement: Figure S2 — IRF3 does not co-localize with MAVS. A549 cells were fixed with 4% paraformaldehyde and incubated with anti-MAVS and anti-IRF3antibodies. MAVS and IRF3 proteins were detected with secondary antibody coupled to Alexa 488 (MAVS, green) or Alexa555 (IRF3, red). (TIF) [file pone.0045136.s002.tif]

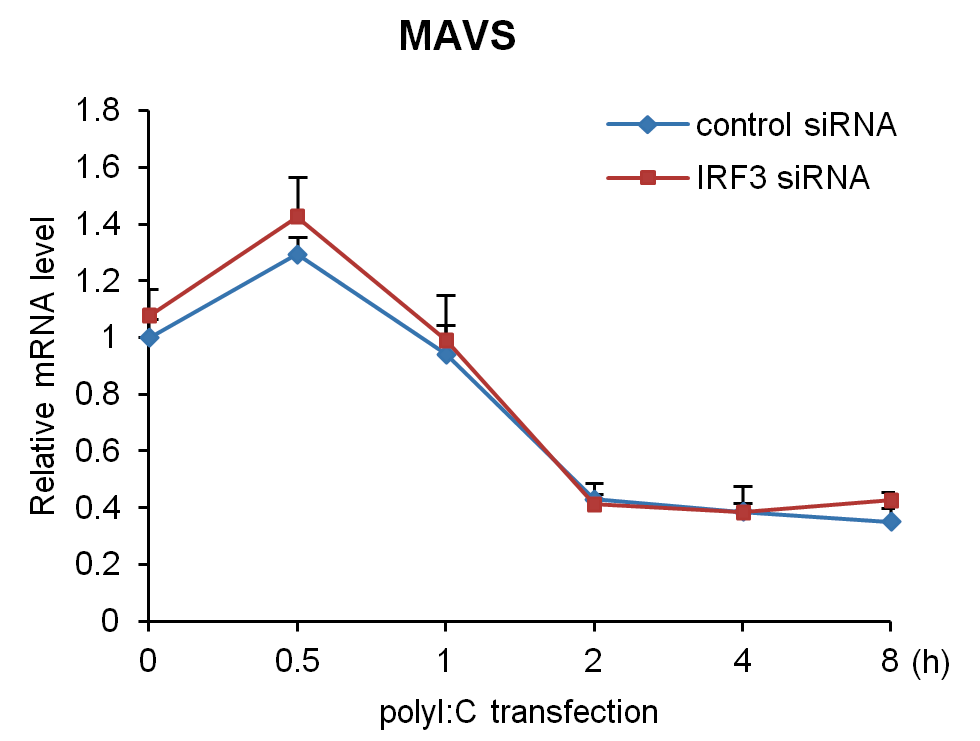

Supplement: Figure S3 — IRF3 does not influence MAVS mRNA decay. Following the knockdown of IRF3 in A549 cells, polyI:C was transfected and the cells were incubated for up to 8 h. Total RNA from the cells was extracted and further analyzed by quantitative RT-PCR. Data are presented as mean ± SD of three independent experiments. (TIF) [file pone.0045136.s003.tif]
